# Postoperative, but Not Preoperative, MELD-3.0 Prognosticates 3-Month Procedural Success in Patients Undergoing Orthotopic Heart Transplantation

**DOI:** 10.3390/jcm13195816

**Published:** 2024-09-28

**Authors:** Jakub Ptak, Mateusz Sokolski, Joanna Gontarczyk, Roksana Mania, Piotr Byszuk, Dominik Krupka, Paulina Makowska, Magdalena Cielecka, Anna Boluk, Mateusz Rakowski, Mateusz Wilk, Maciej Bochenek, Roman Przybylski, Michał Zakliczyński

**Affiliations:** 1 Institute of Heart Diseases, Wroclaw Medical University, Poland Borowska 213, 50-556 Wroclaw, Poland; 2 Clinic of Cardiac Transplantation and Mechanical Circulatory Support, Institute of Heart Diseases, Wroclaw Medical University, 50-556 Wroclaw, Poland; 3 Centre for Heart Diseases, University Hospital, 50-556 Wroclaw, Poland; 4 Student Scientific Club of Transplantology and Advanced Therapies of Heart Failure, Institute of Heart Diseases, Wroclaw Medical University, 50-556 Wroclaw, Poland

**Keywords:** HTx, MELD-3.0, cardio-renal syndrome, cardiohepatic syndrome, hepatopathy, heart failure

## Abstract

**Background/Objectives**: Multi-organ failure (MOF) often complicates advanced heart failure (HF), contributing to a poor prognosis. The Model of End-Stage Liver Disease 3.0 (MELD-3.0) scale incorporates liver and kidney function parameters. This study aims to evaluate the prognostic significance of the MELD-3.0 score in patients with advanced HF who have undergone heart transplantation (HTx). **Methods**: The MELD-3.0 score was computed using the average values of the international normalized ratio and bilirubin, creatinine, sodium, and albumin levels during a hospital stay following HTx. The average MELD-3.0 scores from the period of 1 month preceding HTx and 1 week after HTx were analyzed. The primary endpoint of the study was the 6-month total mortality, and the secondary endpoint was ICU hospitalization time after HTx. **Results**: The analysis included 106 patients undergoing HTx, with a median age of 53 years (44–63), 81% of whom were male. Within 6 months post-HTx, 17 patients (16%) died; those patients had a higher 1-week post-HTx MELD-3.0 score of 18.3 (14.5–22.7) in comparison to survivors, whose average score was 13.9 (9.5–16.4), *p* < 0.01. There was no difference in MELD 3.0 score in the pre-HTx period: 16.6 (11.4–17.8) vs. 12.3 (8.6–17.1), *p* = 0.36. The post-HTx MELD-3.0 score independently predicted death: RR 1.17 (95% CI 1.05–1.30), *p* < 0.01. A Receiver Operating Characteristic (ROC) determined the cut-off value of the MELD-3.0 score as 17.3 (AUC = 0.83; sensitivity—67%; specificity—86%). Survivors with scores above this value had a longer ICU hospitalization time: 7 (5.0–11.0) vs. 12 (8–20) days (*p* = 0.01). **Conclusions**: The post-HTx MELD-3.0 score serves as an independent predictor of an unfavorable prognosis in patients with advanced HF undergoing HTx. The evaluation of MELD-3.0 scores provides additional prognostic information in this population.

## 1. Introduction

Heart failure (HF), particularly in advanced stages, contributes to a cascade of multi-organ failure (MOF), exerting a profound impact on disease progression and adversely influencing outcomes [1,2,3,4]. The intricate interplay between HF and the liver and kidneys gives rise to cardiohepatic and cardio-renal syndromes, further complicating the clinical landscape.

The Model of End-Stage Liver Disease (MELD) was originally designed for prognostic assessment in advanced liver disease [5,6,7,8]. Over time, the MELD scale has undergone iterative updates and refinements, culminating in the latest iteration, MELD-3.0. This advanced scoring system allows for the concurrent evaluation of liver and kidney function, presenting a unique opportunity to prognosticate in patients with HF. Consequently, the markers of liver dysfunction and the MELD scale have assumed significant importance in the risk stratification of HF patients [9,10,11]. Previous versions of the MELD have demonstrated prognostic value, especially in the context of patients undergoing heart transplantation (HTx) [11,12,13,14,15,16,17]. However, the independent prognostic role of the MELD, particularly MELD-3.0, in post-HTx mortality remains underexplored.

Parameters directly connected with the cardiovascular system, such as troponin, have established prognostic value in both chronic or acute HF patients [18,19,20,21] and those undergoing HTx [22,23]. Universal biomarkers like hemoglobin (Hb) and C-reactive protein (CRP) levels have also demonstrated their predictive utility in patients undergoing surgery [24,25,26,27,28].

## 2. Materials and Methods

This study aims to evaluate the prognostic significance of the MELD-3.0 score in patients with advanced HF who have undergone HTx. Beyond this, the objective is to establish that the assessment of MOF severity using the MELD-3.0 scale provides additional and independent prognostic information compared to traditional markers. Through this exploration, we seek to contribute to a more nuanced understanding of risk stratification in HF patients undergoing HTx. The primary endpoint of this study was the 6-month total mortality, and the secondary endpoint was the intensive care unit (ICU) hospitalization time after HTx.

This study was conducted in the Institute of Heart Diseases, University Hospital of Wroclaw, Poland, and progressed through a structured series of phases. Initially, essential laboratory parameter values were retrospectively entered into a database. Subsequently, the averages of these parameter values were computed, encompassing the period of both 1 month before HTx and 1 week during the hospital stay following the HTx procedure. In the third phase, the MELD-3.0 score was calculated based on the derived average values. Finally, the amassed dataset underwent rigorous analysis in the fourth phase.

The study cohort comprised patients who underwent HTx between 25 February 2021 and 26 June 2023, amounting to a total of 107 individuals. Exclusion criteria were applied to patients lacking the complete set of parameters necessary for MELD-3.0 score calculation, resulting in the exclusion of 1 patient. Throughout their hospitalization, each patient underwent standard clinical evaluation and received recommended treatments. The 6-month follow-up data were collected as part of the protocolar hospital stay, aligning with the guidelines set forth by the International Society for Heart and Lung Transplantation [29]. A total of 13 patients with HF had indications to anticoagulation therapy with novel oral anticoagulants (NOACs) or vitamin K antagonists (VKAs) before HTx. We decided not to exclude them from our analysis because their international normalized ratio (INR) did not differ significantly from those of patients that were not on VKAs (*p* > 0.5). No patients were treated with VKAs after the HTx procedure.

The MELD-3.0 score was calculated using the following formula:

1.33 (if woman) + 4.56 × ln[bilirubin] + 0.82 × (137 − [Na]) − 0.24 × (137 − [Na]) × ln[bilirubin] + 9.09 × ln(INR) + 11.14 × ln[creatynine] + 1.85 × (3.5 − [albumin]) − 1.83 × (3.5 − [albumin]) × ln[creatynine] + 6.

Following the recommendations of the United Network for Organ Sharing for liver transplant organ allocation in the USA and other authors that created the score, the lower limit of bilirubin and creatinine was set at 1.0 mg/dL [11,30]. In practice, if a patient had creatinine or bilirubin levels lower than 1.0 mg/dL, the value of 1.0 mg/dL was used in the calculations to prevent negative values in the formula.

Normally distributed continuous variables were reported as means ± standard deviation. For variables exhibiting a skewed distribution, medians along with lower and upper quartiles were employed. The normality of the variable distribution was assessed using the Shapiro–Wilk test. To explore intergroup differences, the T-test was applied for normally distributed variables, while the Mann–Whitney U test was employed for variables with a skewed distribution, considering the non-parametric nature of the latter. Categorical variables were succinctly presented as numbers accompanied by percentages. Differences in categorical variables between the compared groups were assessed using the chi-square test.

The primary endpoint of this study was focused on all-cause mortality at the 6-month follow-up. To analyze the prognostic factors associated with mortality, Cox proportional hazards regression models were employed, calculating the risk ratio (RR) alongside the corresponding 95% confidence interval (95% CI). The models incorporated the MELD-3.0 score, derived from the average laboratory parameter levels throughout the analyzed period, alongside established prognostic markers like troponins, CRP, Hb, and two other parameters: Potassium (K) and procalcitonin (PCT).

Additionally, a Receiver Operating Characteristic (ROC) curve was constructed for MELD-3.0 alone, and a cut-off value was determined. The Mann–Whitney U test was utilized to compare the duration of the ICU hospitalization time between patient groups above and below the MELD-3.0 cut-off value.

In this study, meticulous attention was given to follow-up data, and no loss was reported. However, to ensure the accuracy of the MELD-3.0 score calculations, patients with missing data crucial for this computation were deliberately excluded from this study. It is noteworthy that any remaining missing data are transparently presented in the accompanying tables, providing a comprehensive overview of the study population.

A significance threshold of *p* < 0.05 was applied. All statistical analyses were conducted using the STATISTICA data analysis software system (StatSoft Polska Sp. z o.o. 2024. Zestaw Plus version 5.0.96. www.statsoft.pl).

## 3. Results

### 3.1. Participants

A total of 106 patients with advanced HF treated with HTx were included. Within 6 months post-HTx, 17 patients (16%) died. The primary cause of mortality was MOF, primarily attributed to postoperative HF (*n* = 9). Other contributing factors included sepsis (*n* = 3) and hemorrhage (*n* = 2). All 3-month survivors successfully completed the prescribed protocolar follow-up. The median time of death was 14 days, mean—30 days after the procedure.

### 3.2. Descriptive Data

The majority of the cohort was men (81%), with a median age of 53 (44–63) years, 47 (44%) of whom were diagnosed with hypertension, 27 (25%) with diabetes, and 32 (30%) with chronic kidney disease. The most common HF etiology was ischemic (43%). The mean central venous pressure (CVP) was 10.7 (±6.2) mmHg, mean pulmonary artery pressure (PAM) 30.3 (±10.1) mmHg, median cardiac output (CO) measured with the thermodilution technique 4,1 L/min (3.5–4.7), and pulmonary vascular resistance (PVR) 2.3 (1.8–3.2) WU, Table 1.

The median NT-proBNP level was 4276 (2130–9847) pg/mL, creatinine 1.11 (0.94–1.41) mg/dL, bilirubin 1.28 (0.82–2.05) mg/dL, INR 1.27 (1.11–1.67), Hb 12.77 (10.63–14.23) g/dL, albumin 3.5 (2.9–4.0) g/dL, CRP 13.7 (3.5–55.5) mg/L, Natrium (Na) 138.6 (136.4–139.9) mmol/L, and troponin 35.8 (13.6–1106) ng/L. The median MELD-3.0 score before HTx was 12.8 (8.9–17.4) (Table 2).

Over a six-month observation period, this study documented a total of 17 fatalities within the cohort. Notably, among the survivors of this period, 33 individuals required renal replacement therapy during their hospital stay following HTx.

### 3.3. Main Results

The MELD-3.0 score assessed based on data from 1 month before HTx did not show a statistically significant difference between the groups of the deceased and survivors (*p* > 0.36) (Table 2).

Table 3 presents the differences in laboratory parameters one week after HTx between the groups of 6-month survivors and deceased individuals.

After adjusting for the aforementioned parameters, the MELD-3.0 score from the first week of the hospital stay after HTx emerged as a significant predictor of death: (RR 1.17 (95% CI 1.05–1.30), *p* < 0.01) (Table 4).

The MELD-3.0 score one week post-HTx remained an independent predictor of death in various proportional hazards regression models adjusted for troponin, Hgb, K, CRP, or PCT (Table 5).

### 3.4. Other Analyses

**Figure 1:**
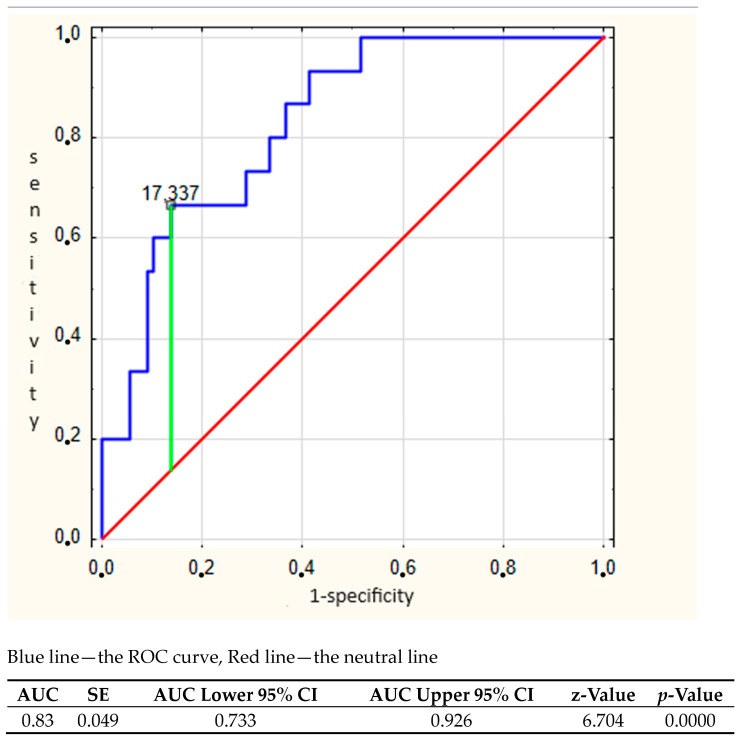
ROC curve for MELD 3.0 scores 1 month after HTx.

The constructed ROC curve for the MELD-3.0 score based on data from the first month after HTx suggested a cut-off value of 17.3 (AUC = 0.83; sensitivity—67.8%; specificity—86.2%) (Figure 1).

When the 3-month follow-up survivors were divided into groups based on the aforementioned MELD-3.0 score cut-off value, a statistically significant difference of 5 days in ICU hospitalization time was observed: 7 (5.0–11.0) vs. 12 (8–20) days, (*p* = 0.01) (Table 6).

## 4. Discussion

Our study demonstrates a 6-month survival rate of 84% for heart transplant (HTx) recipients, with a median age of 53 years (44–62 years). This aligns closely with data from the International Registry of Thoracic Organ Transplants (TTX), which reports a 6-month survival rate in Europe of approximately 83% for recipients aged 40–59 years and around 78% for those aged 60 years and older [31]. In our cohort, over 33% of HTx patients were aged 60 years or more. According to the 2023 International Society for Heart and Lung Transplantation (ISHLT) report, the median HTx recipient age is 54 years, with 77.9% being male, while the median BMI is 25.7 kg/m^2^, and the median eGFR is 75.3 mL/min/1.73 m^2^ [32]. Our HTx cohort is slightly younger (median age of 53 years) and has a higher mean BMI (26 kg/m^2^), with a similar gender distribution. The proposed MELD 3.0 score cut-off values, ranging between 14 and 20, are consistent with our findings [33,34].

The MELD score has undergone validation and is widely employed for risk assessment in patients with advanced liver disease [1,2,3]. Combining data on liver and kidney function, the score holds potential for assessing patients with HF, impacting both organs. The validation of its newest version, MELD-3.0, as a marker of hepato-renal conditions in the advanced HF population remains incomplete. However, the well-documented prognostic value of its previous versions in HF and HTx is established [9,10,11,12,13,14,15,16,17]. We posit that MOF resulting from advanced HF is a major contributor to a poor prognosis. Numerous reports detail individual organ dysfunctions and their clinical implications. Recently, attention has shifted towards understanding the interaction between organs and MOF in advanced HF, offering a more nuanced view of HF pathophysiology. Common pathways may underlie the injury and dysfunction of end-organs.

Our study was conducted exclusively on patients undergoing HTx at the University Hospital in Wroclaw, with a 6-month follow-up to ensure data completeness. As a result, this study could not evaluate the long-term prognostic value of the MELD-3.0 score, though such information would be highly valuable. The study design involved a meticulous process, from retrospective data collection to the calculation of the MELD-3.0 score and subsequent rigorous analysis. The cohort consisted of 106 HTx patients, and the findings revealed that the MELD-3.0 score during post-HTx hospitalization, when adjusted for other established prognostic factors, was a significant predictor of mortality. To our knowledge, this study is the first to show that impaired hepato-renal function, as indicated by an elevated MELD-3.0 score during hospitalization, independently predicts prognosis in advanced HF patients post-HTx. This highlights the potential role of the MELD-3.0 score in guiding therapeutic decisions, such as treatment escalation or discharge planning.

To mitigate potential bias, this study encompassed all patients who underwent HTx at our center. A comprehensive analysis was conducted, encompassing not only laboratory parameters both pre- and post-HTx but also demographic and comorbidity profiles. This exhaustive examination aimed to eliminate any inherent disparities between the groups of 6-month survivors and deceased patients at the baseline, ensuring a robust and unbiased foundation for subsequent analyses.

It is possible that the INR of patients who discontinued VKA administration shortly before qualification for HTx might be slightly higher than expected based on liver function. Nevertheless, VKAs were discontinued in the qualification process; the patients awaiting the procedure in the hospital were treated with heparin. Additionally, there was no statistically significant difference in the INR between patients on VKAs prior to the procedure and those who were not (*p* > 0.5). After HTx, we found no indications for VKA or NOAC in any patient. Low-molecular weight heparin was often used in prophylactic doses in the first weeks after HTx. The primary limitation appears to be the differences between the groups of 6-month survivors and the deceased in PAM and CVP (approaching statistical significance) before HTx. Both may be attributed to overhydration, potentially impairing liver and kidney function represented by the MELD-3.0 score. Conversely, other liver and kidney laboratory parameters did not show significant differences between these groups. This leads us to believe that the impacts of PAM and CVP on postoperative MELD-3 score values were limited.

Despite comprehensive efforts to minimize bias, this study recognizes its limitations, such as the restriction of the dataset to patients from a specific hospital and the inability to assess long-term prognostic value due to the chosen 6-month follow-up.

Our innovative approach involved using the mean values of laboratory parameters from predefined time intervals rather than single-point values. This decision aimed to minimize the risk of false lab results influencing our analysis. Moreover, we contend that the mean values from the time interval more accurately reflect organ condition and function than individual lab tests. We believe that this approach may be used more frequently in future data analyses.

It is conceivable that physicians may be hesitant to calculate such a complex score to obtain risk assessments in HTx patients. From our perspective, however, this score presents a significant opportunity to streamline MOF assessment. Instead of considering a myriad of laboratory parameters focused on individual organs, physicians could input the data into a pre-established formula, resulting in easily interpretable outcomes. Notably, the components comprising the MELD-3.0 score are likely to be assessed in HTx patients as part of routine clinical practice. This approach could enhance efficiency and facilitate a more standardized evaluation of organ function in HTx patients.

## 5. Conclusions

The results suggest that an elevated MELD-3.0 score, reflecting impaired hepato-renal function, independently predicts mortality in this patient population. This score could simplify the complex assessment of MOF in advanced HF, potentially improving clinical outcomes through more standardized and efficient evaluations.

## Figures and Tables

**Table 1:** Characteristics of population. Data obtained before heart transplantation.

Parameter	All Patients (*n* = 106)	Deceased (*n* = 17)	6-Month Survivors (*n* = 89)	*p*-Value
Age, years (SD)	52.1 (12.6)	51.4 (15.7)	52.2 (12.0)	0.81
BMI, kg/m^2^ (SD)	26.0 (4.0)	26.7 (5.1)	25.9 (3.8)	0.53
Body mass, kg (SD)	77.9 (15.2)	76.2 (21.0)	78.1 (14.1)	0.65
Ischemic HF etiology, *n* (%)	46 (43)	8 (50)	38 (42)	0.54
Sex, male (%)	87 (82)	14 (88)	73 (81)	0.91
Hypertension, *n* (%)	47 (44)	7 (44)	40 (44)	0.81
Hyperlipidemia, *n* (%)	42 (39)	5 (31)	37 (41)	0.37
DM, *n* (%)	27 (25)	4 (25)	23 (26)	0.87
CKD, *n* (%)	35 (33)	4 (25)	31 (34)	0.53
CVP, mmHg (SD)	10.7 (6.2)	13.6 (6.2)	10.0 (6.0)	0.06
PAM, mmHg (SD)	30.3 (10.1)	36.9 (11.0)	29.0 (9.4)	0.01
PCWP, mmHg (SD)	20.1 (6.4)	22.0 (4.3)	20.0 (6.7)	0.27
CO, L/min (IQR)	4.1 (3.5–4.7)	4.3 (3.8–4.8)	4.0 (3.4–4.5)	0.18
PVR, WU (IQR)	2.3 (1.8–3.2)	2.5 (2.3–3.8)	2.2 (1.7–3.0)	0.06
Hospitalization time, days (IQR)	38.0 (26.0–62.0)	29.0 (17.0–52.0)	41.0 (28.0–61.0)	0.10
ICU hospitalization time (IQR)	7.0 (5.0–13.0)	12.0 (3.0–18.0)	7.0 (6.0–12.0)	0.47
MCS pre-HTx, *n* (%)	26 (26)	5 (31)	21 (23)	0.59

CVP—central venous pressure, SD—standard deviation, IQR—interquartile range, PAM—pulmonary artery mean pressure, PCWP—pulmonary capillary wedge pressure, CO—cardiac output, PVR—pulmonary vascular resistance, ICU—intensive care unit, BMI—body mass index, HF—heart failure, DM—diabetes mellitus, CKD—chronic kidney disease, MCS—mechanical circulatory support.

**Table 2:** Average laboratory parameters—1 month before HTx.

Parameter	Reference Range	All Patients (*n* = 106)	Deceased (*n* = 17)	6-Month Survivors (*n* = 89)	*p*-Value
MELD 3.0 (IQR)	-	12.8 (7.9–17.4)	16.6 (11.4–17.8)	12.3 (8.6–17.1)	0.36
WBC, k/µL (IQR)	4–10	9.18 (6.98–12.27)	9.71 (6.85–16.45)	9.17 (6.98–12.17)	0.57
Hgb, g/dL (IQR)	14–18/12–16	12.9 (11.3–14.2)	12.0 (9.9–13.5)	12.9 (11.7–14.3)	0.07
Hct, % (IQR)	40–54/37–47	38.5 (34.1–42.3)	37.1 (30.3–41.0)	37.3 (32.4–41.6)	0.17
Plt, k/µL (IQR)	140–440	197.6 (151.9–241.7)	185.0 (170.3–281.9)	199.1 (153.4–249.4)	0.70
INR (IQR)	0.8–1.2	1.29(1.11–1.56)	1.24 (1.12–1.82)	1.28 (1.11–1.55)	0.62
APTT, s (IQR)	25.4–36.9	37.9 (32.9–46.2)	38.4 (33.1–42.6)	37.4 (35.4–45.9)	0.98
D-Dimer, mg/L (IQR)	0–0.5	1.97(1.13–5.76)	3.04 (1.50–5.89)	1.93 (1.09–5.73)	0.21
Bilirubin, mg/dL (IQR)	0.2–1.2	1.23 (0.80–2.07)	1.40 (0.90–1.90)	1.19 (0.80–2.05)	0.56
ALAT, IU/L (IQR)	0–45	29.0 (21.0–47.9)	31.8 (19.9–93.1)	28.1 (21.0–46.6)	0.62
Aspat, IU/L (IQR)	0–35	40.3 (23.9–59.0)	33.3 (26.3–85.8)	39.7 (23.0–59.8)	0.88
ALP, IU/L (IQR)	40–150	87.0 (62.0–103.0)	91.7 (70.0–142.3)	88.0 (62.3–102.6)	0.41
CK, IU/L (IQR)	0–171	198.4 (63.5–457.0)	105.3 (33.5–998.0)	198.4 (63.5–449.5)	0.73
Glucose, mg/dL (IQR)	70–99	105.2 (95.0–121.3)	107.5 (88.3–124.7)	105.2 (95.0–121.0)	0.94
Creatinine, mg/dL (IQR)	0.6–1.3	1.10 (0.94–1.39)	1.23 (1.07–1.42)	1.10 (0.94–1.39)	0.40
Uric acid, mg/dL (IQR)	3.5–7.2	6.07 (5.18–8.40)	7.25 (5.70–8.03)	6.13 (5.18–8.40)	0.51
Total protein, g/dL (SD)	6.6–8.3	6.66 (0.97)	6.48 (0.99)	6.68 (0.98)	0.61
Albumin, g/dL (IQR)	3.5–5.2	3.60 (3.00–4.10)	3.10 (2.88–3.43)	3.60 (3.00–4.12)	0.03
CRP, mg/L (IQR)	0–5	10.3 (3.3–53.9)	17.8 (5.2–58.0)	10.3 (3.2–52.2)	0.37
LDL, mg/dL (IQR)	0–135	69.0 (41.0–78.0)	63.5 (51.0–72.0)	66.6 (43.5–76.5)	0.84
TG, mg/dL (IQR)	0–150	101.5 (85.0–132.0)	114.0 (84.0–136.0)	101.5 (85.0–132.0)	0.77
Na, mmol/L (IQR)	136–146	138.9 (136.4–140.0)	138.0 (135.6–139.9)	138.8 (136.4–139.9)	0.37
K, mmol/L (IQR)	3.5–5.1	4.32 (4.14–4.55)	4.20 (3.91–4.37)	4.32 (4.14–4.55)	0.13
PCT, ng/L (IQR)	0–0.05	0.12 (0.04–0.42)	0.23 (0.07–0.91)	0.12 (0.04–0.40)	0.19
Troponin, ng/L (IQR)	0–34.2	30 (13–1104)	291 (16–7554)	28 (12–656)	0.06
NTproBNP, pg/mL (IQR)	0–125	4276 (2158–8180)	3346 (1933–9246)	4244 (2135–8180)	0.84

MELD—model for end-stage liver disease, WBC—white blood count, Hgb—hemoglobin, Hct—hematocrit, Plt—platelets, INR—international normalized ratio, APTT—Activated Partial Thromboplastin Clotting Time, ALAT—alanine aminotransferase, Aspat—aspartate aminotransferase, ALP—Alkaline Phosphatase, CK—Creatine Kinase, CRP—C-reactive protein, LDL—low-density lipoprotein, TG—triglyceride, PCT—procalcitonin, NTproBNP—N-terminal pro B-type natriuretic peptide, IQR—interquartile range, SD—standard deviation.

**Table 3:** Average laboratory parameters—1 week after HTx.

Parameter	Reference Range	All Patients (*n* = 106)	Deceased (*n* = 17)	6-Month Survivors (*n* = 89)	*p*-Value
MELD 3.0 (IQR)	-	14.5 (10.1–18.3)	18.3 (14.5–22.7)	13.9 (9.5–16.4)	0.01
WBC, k/µL (IQR)	4–10	17.4 (14.0–21.0)	23.2 (16.2–28.1)	17.3 (13.4–20.0)	0.02
Hgb, g/dL (IQR)	14–18/12–16	10.0 (9.5–10.5)	9.70 (9.23–10.43)	10.1 (9.5–10.5)	0.57
Plt, k/µL (IQR)	140–440	133.4 (101.0–182.0)	103.0 (79.8–137.0)	138.0 (107.5–187.0)	0.06
INR (IQR)	0.8–1.2	1.19 (1.09–1.30)	1.33 (1.22–1.68)	1.16 (1.09–1.27)	0.00
APTT, s (IQR)	25.4–36.9	34.0 (31.7–37.6)	39.8 (34.0–45.2)	33.5 (31.6–37.1)	0.03
D-Dimer, mg/L (IQR)	0–0.5	1.10 (0.73–1.81)	1.83 (1.25–4.39)	1.01 (0.69–1.58)	0.00
Bilirubin, mg/dL (IQR)	0.2–1.2	1.30 (0.80–2.10)	2.70 (1.40–5.55)	1.18 (0.70–1.85)	0.00
ALAT, IU/L (IQR)	0–45	33.8 (25.5–29.3)	119.3 (42.5–675.5)	32.0 (24.3–50.8)	0.00
Aspat, IU/L (IQR)	0–35	55.5 (35.0–87.0)	139.0 (58.0–480.0)	50.0 (33.0–74.0)	0.00
ALP, IU/L (IQR)	40–150	75.0 (57.0–100.0)	93.0 (71.0–104.0	74.5 (53.0–98.5)	0.27
CK, IU/L (IQR)	0–171	536.0 (170.0–799.0)	871 (594–1024)	439.5 (135.0–789.0)	0.00
Glucose, mg/dL (IQR)	70–99	119.0 (92.5–144.0)	133.0 (113.8–176.0)	115.5 (90.5–142.0)	0.16
Creatinine, mg/dL (IQR)	0.6–1.3	1.44 (1.06–1.80)	1.41 (1.19–1.71)	1.44 (1.05–1.81)	0.63
Albumin, g/dL (IQR)	3.5–5.2	3.2 (3.0–3.4)	2.98 (2.78–3.18)	3.20 (3.00–3.40)	0.02
CRP, mg/L (IQR)	0–5	48.3 (29.6–68.7)	56.3 (48.0–97.2)	46.9 (29.5–66.4)	0.38
Na, mmol/L (IQR)	136–146	137.0 (136.0–140.0)	137.0 (136.0–140.0)	137.0 (136.0–140.0)	0.44
K, mmol/L (IQR)	3.5–5.1	4.50 (4.20–4.80)	4.70 (4.60–4.85)	4.48 (4.12–4.70)	0.02
PCT, ng/L (IQR)	0–0.05	2.62 (0.73–6.78)	6.4 (3.9–28.8)	2.07 (0.65–5.34)	0.01
Troponin, ng/L (IQR)	0–34.2	8116 (4616–14,684)	15,406 (11,102–20,682)	7095 (4366–12,037)	0.00
NTproBNP, pg/mL (IQR)	0–125	10,351 (5485–17,585)	19,551 (9002–28,130)	9485 (4781–15,800)	0.21

MELD—model for end-stage liver disease, WBC—white blood count, Hgb—hemoglobin, Plt—platelets, INR—international normalized ratio, APTT—Activated Partial Thromboplastin Clotting Time, ALAT—alanine aminotransferase, Aspat—aspartate aminotransferase, ALP—Alkaline Phosphatase, GGTP—Gamma Glutamyl Transpeptidase, CK—Creatine Kinase, CRP—C-reactive protein, LDL—low-density lipoprotein, TG—triglyceride, PCT—procalcitonin, NTproBNP—N-terminal pro B-type natriuretic peptide, IQR—interquartile range, SD—standard deviation, RRT—renal replacement therapy.

**Table 4:** Proportional hazards regression model—1 week post-HTx.

*n* = 94, Chi2 = 15.03 *p* = 0.010	Beta	Standard Error	Beta 95% Lower	Beta 95% Upper	*p*-Value	Risk Ratio	Risk Ratio 95% Lower	Risk Ratio 95% Upper
Troponin, ng/L	0.00	0.00	0.00	0.00	0.15	1.00	1.00	1.00
Hgb, g/dL	−0.15	0.39	−0.90	0.61	0.71	0.86	0.41	1.84
MELD-3.0	0.16	0.06	0.05	0.27	0.01	1.17	1.05	1.30
K, mmol/L	0.63	1.01	−1.36	2.61	0.54	1.87	0.26	13.65
CRP, mg/L	0.01	0.01	−0.01	0.02	0.24	1.01	0.99	1.02

Hgb—hemoglobin, CRP—C-reactive protein, MELD—model for end-stage liver disease.

**Table 5:** Proportional hazards regression model—1 week post-HTx.

Covariates of Proportional Hazards Regression Model									
1	2	3	4	5	*p*-Value of Model	*p*-Value of MELD-3.0	Risk Ratio	95% Lower Confidence Interval	95% Upper Confidence Interval
+	−	−	−	−	0.001	0.002	1.19	1.07	1.33
−	+	−	−	−	0.001	0.044	1.11	1.01	1.24
−	−	+	−	−	0.002	0.002	1.17	1.06	1.29
−	−	−	+	−	0.001	0.003	1.20	1.06	1.34
−	−	−	−	+	0.001	0.005	1.17	1.05	1.31
+	−	+	−	−	0.004	0.002	1.19	1.07	1.33
+	−	−	+	−	0.005	0.002	1.19	1.07	1.32
+	−	−	−	+	0.002	0.005	1.17	1.05	1.31
−	+	+	−	−	0.001	0.044	1.11	1.01	1.24
−	+	−	+	−	0.001	0.042	1.12	1.01	1.24
−	+	−	−	+	0.000	0.209	1.08	0.96	1.21
−	−	+	+	−	0.008	0.001	1.19	1.07	1.33
−	−	+	−	+	0.003	0.005	1.17	1.05	1.31
−	−	−	+	+	0.008	0.001	1.19	1.07	1.33
+	−	+	+	−	0.01	0.002	1.19	1.07	1.32
+	−	+	−	+	0.003	0.005	1.17	1.05	1.31
+	−	−	+	+	0.004	0.005	1.17	1.05	1.31
−	+	+	+	−	0.01	0.046	1.11	1.01	1.24
−	+	+	−	+	0.001	0.185	1.08	0.96	1.21
−	+	−	+	+	0.001	0.218	1.08	0.96	1.21
−	−	+	+	+	0.008	0.005	1.17	1.05	1.31
+	−	+	+	+	0.01	0.006	1.17	1.05	1.30
−	+	+	+	+	0.001	0.210	1.08	0.96	1.21

“+” —covariate included to the regression model. “−” —covariate not included to the regression model. 1—CRP—C-reactive protein. 2—PCT—procalcitonin. 3—Hb—hemoglobin. 4—K—Potassium. 5—troponin.

**Table 6:** Hospitalization time based on ROC cut-off value = 17.3.

Parameter	All Patients (*n* = 90)	MELD-3 < 15.1 (*n* = 75)	MELD-3 > 15.1 (*n* = 15)	*p*-Value
Hospitalization time, days (IQR)	41.5 (28.0–65.0)	38.0 (26.0–61.0)	55.0 (36.0–85.0)	0.07
ICU hospitalization time (IQR)	7.0 (6.0–12.0)	7.0 (5.0–11.0)	12.0 (8.0–20.0)	0.01

MELD—model for end-stage liver disease, IQR—interquartile range, ICU—intensive care unit.

## Data Availability

All of the obtained data is presented in the tables and figures.
